# Tetra­aqua­diazido­cobalt(II) 4,4′-dicarboxyl­ato-1,1′-ethyl­enedi­pyridinium dihydrate

**DOI:** 10.1107/S1600536809049848

**Published:** 2009-11-25

**Authors:** Kun Wang, Yan-Qin Wang, Jian-Yong Zhang, En-Qing Gao

**Affiliations:** a Shanghai Key Laboratory of Green Chemistry and Chemical Processes, Department of Chemistry, East China Normal University, Shanghai 200062, People’s Republic of China

## Abstract

In the title compound, [Co(N_3_)_2_(H_2_O)_4_]·C_14_H_12_N_2_O_4_·2H_2_O, the metal complex mol­ecule is centrosymmetric, the Co(II) ion being six-coordinated by two azide N atoms and four aqua O atoms with a *trans*-octa­hedral geometry. The zwitterionic organic mol­ecule is also centrosymmetric. In the crystal, the components are associated into a two-dimensional network through O—H⋯O hydrogen bonds. Further O—H⋯O and O—H⋯N inter­actions give a three-dimensional structure. The free water molecule is disordered over two positions in a 0.787 (5):0.213 (5) ratio.

## Related literature

For background information on hydrogen bonds in crystal engineering, see: Baures *et al.* (2006 ▶); Braga & Grepioni (2000 ▶); Maly *et al.* (2006 ▶). For the ligand synthesis, see: Loeb *et al.* (2006 ▶). For hydrogen-bond motifs, see: Bernstein *et al.* (1995 ▶); Etter (1990 ▶).
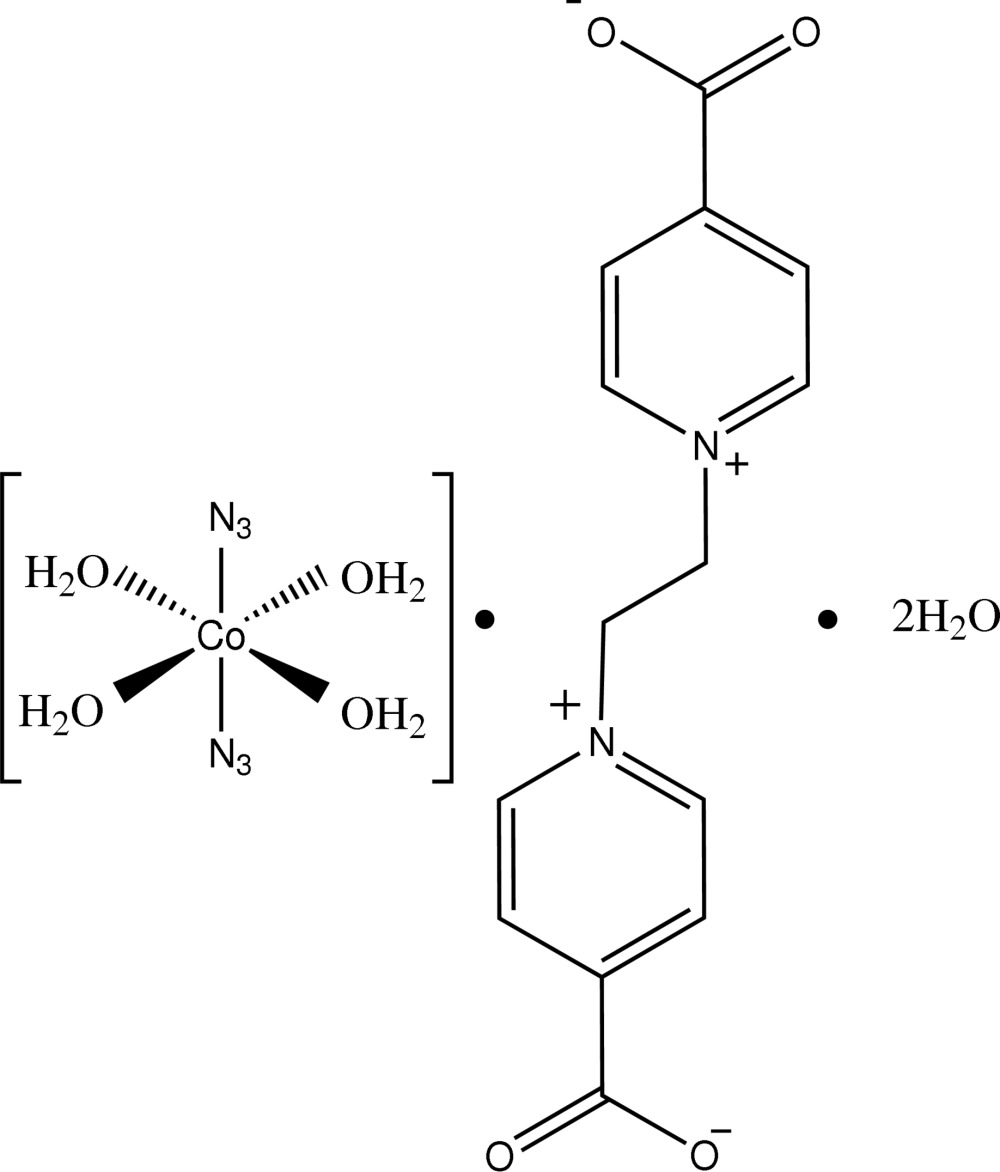


## Experimental

### 

#### Crystal data

[Co(N_3_)_2_(H_2_O)_4_]·C_14_H_12_N_2_O_4_·2H_2_O
*M* *_r_* = 523.34
Triclinic, 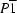

*a* = 7.1951 (5) Å
*b* = 9.0354 (7) Å
*c* = 9.0915 (5) Å
α = 71.402 (3)°
β = 85.568 (2)°
γ = 69.752 (2)°
*V* = 525.20 (6) Å^3^
*Z* = 1
Mo *K*α radiation
μ = 0.89 mm^−1^
*T* = 296 K
0.08 × 0.08 × 0.02 mm

#### Data collection

Bruker APEXII CCD area-detector diffractometer
Absorption correction: multi-scan (*SADABS*; Bruker, 2007 ▶) *T*_min_ = 0.932, *T*_max_ = 0.982
6498 measured reflections
2029 independent reflections
2016 reflections with *I* > 2σ(*I*)
*R*_int_ = 0.020

#### Refinement

*R*[*F* ^2^ > 2σ(*F* ^2^)] = 0.027
*wR*(*F* ^2^) = 0.089
*S* = 1.18
2029 reflections
183 parameters
13 restraints
H atoms treated by a mixture of independent and constrained refinement
Δρ_max_ = 0.34 e Å^−3^
Δρ_min_ = −0.35 e Å^−3^

### 

Data collection: *APEX2* (Bruker, 2007 ▶); cell refinement: *SAINT* (Bruker, 2007 ▶); data reduction: *SAINT*; program(s) used to solve structure: *SHELXS97* (Sheldrick, 2008 ▶); program(s) used to refine structure: *SHELXL97* (Sheldrick, 2008 ▶); molecular graphics: *SHELXTL* (Sheldrick, 2008 ▶); software used to prepare material for publication: *SHELXTL*.

## Supplementary Material

Crystal structure: contains datablocks I, global. DOI: 10.1107/S1600536809049848/bg2309sup1.cif

Structure factors: contains datablocks I. DOI: 10.1107/S1600536809049848/bg2309Isup2.hkl

Additional supplementary materials:  crystallographic information; 3D view; checkCIF report

## Figures and Tables

**Table 1:** Hydrogen-bond geometry (Å, °)

*D*—H⋯*A*	*D*—H	H⋯*A*	*D*⋯*A*	*D*—H⋯*A*
O3—H31⋯O5^i^	0.826 (16)	1.908 (18)	2.727 (2)	170 (2)
O3—H31⋯O5′^i^	0.826 (16)	1.96 (2)	2.664 (8)	143 (3)
O4—H41⋯O2^ii^	0.812 (16)	2.072 (18)	2.870 (2)	167 (3)
O5—H51⋯O2^iii^	0.802 (17)	2.110 (18)	2.877 (3)	160 (4)
O3—H32⋯O1	0.847 (16)	1.847 (17)	2.694 (2)	178 (3)
O4—H42⋯O2	0.846 (16)	1.847 (17)	2.687 (2)	172 (2)
O5—H5⋯N4	0.824 (17)	2.19 (2)	2.864 (3)	138.6 (19)

Symmetry codes: (i) 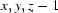; (ii) 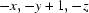; (iii) 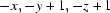.

**Fig. 1.**
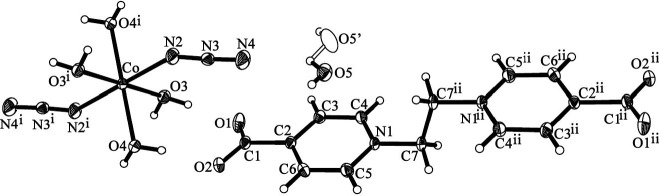
Molecular structure of (I), showing the atom-numbering scheme. Displacement ellipsoids are drawn at the 30% probability level.

**Fig. 2.**
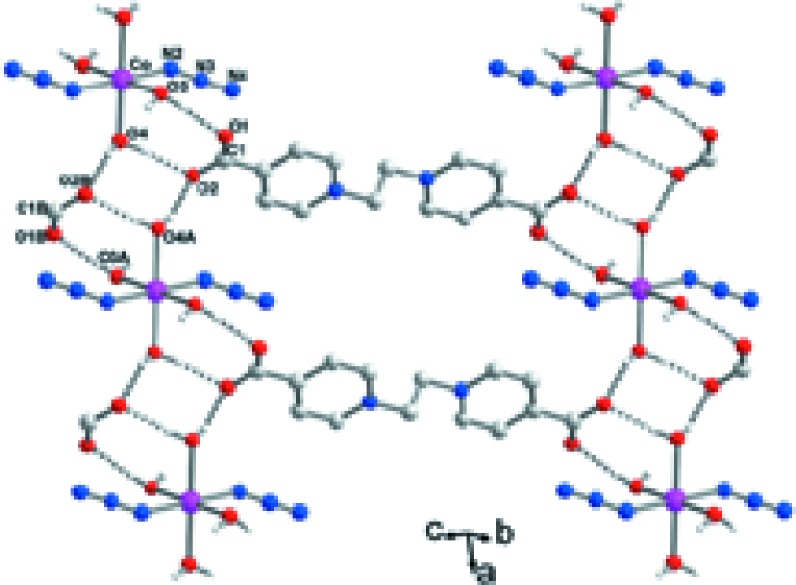
Part of the crystal structure of (I), showing the formation of the two-dimensional network by O—H···O hydrogen bonds which are shown in dashed lines. Hydrogen atoms not involved in the hydrogen bonds have been omitted for clarity.

**Fig. 3.**
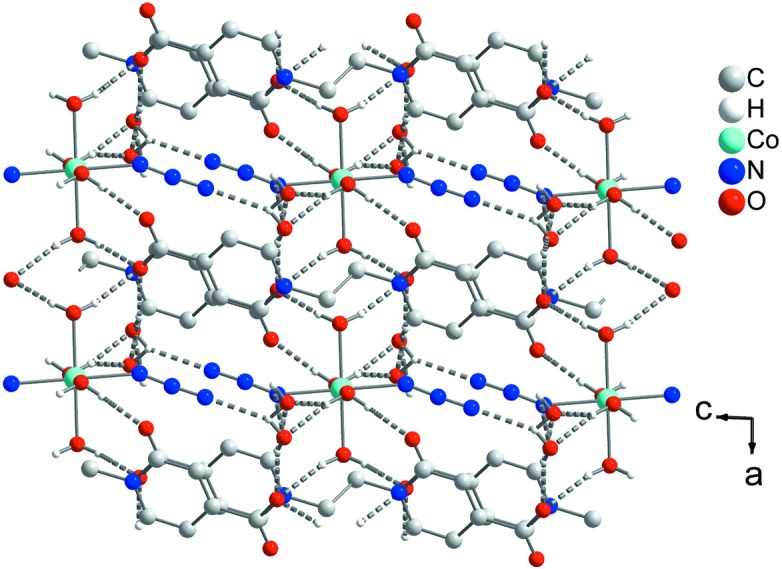
The three-dimensional structure of (I) formed via O—H···O and O—H···N hydrogen-bonds which are shown as dashed lines.

## supplementary crystallographic information

### Comment

The D—H···A hydrogen bonds, ranging from the strong ones involving O—H and N—H to the weak ones involving C—H, have been widely used as a putative tool for engineering organic and metal-organic solids (Braga & Grepioni, 2000; Baures *et al.*, 2006; Maly *et al.*, 2006). In this paper, we report the hydrogen-bonded structure of the title compound, (I), which contains a nuetral metal complex molecule, [Co(N_3_)_2_(H_2_O)_4_], and a zwitterionic dicarboxylate, 1,2-bis(4-carboxylatopyridinium)ethane(Loeb *et al.*, 2006).

The molecular structure is shown in Fig. 1. The metal complex molecule is centrosymmetric, with the Co(II) ion being six-coordinated by two azides and four aquas with a *trans*-octahedral geometry.The axial Co–N distances are slightly shorter than the equatorial Co–O ones. The zwitterionic molecule is also centrosymmetric. As shown in Fig. 2, the inorganic complex molecules and the organic molecules are associated into a two-dimensional sheet along the [101] direction through O—H···O hydrogen bonds involving the coordinated aqua ligands (O3 and O4) and the carboxylate oxygen atoms (O1 and O2). Two O4 aqua ligands from different complex molecules and two O2 atoms from different organic molecules, form a hydrogen-bonded ring which can be denoted by the graph set *R*_4_^2^(8) (Bernstein *et al.*, 1995; Etter, 1990), and the carboxylate group forms a *R*_2_^2^(8) hydrogen-bonded ring with two aqua ligands from the same complex molecule. The three-dimensional structure is formed *via* the hydrogen bonds between the disordered free water molecules (O5 and O5') and the terminal azide nitrogen (N4), the carboxylate oxygen (O2) or the coordinated water molecule (O3) (Fig. 3).

### Experimental

The crystals was synthesized using the hydrothermal method in a 23 ml Teflon-lined Parr bomb. CoCl_2_.6H_2_O (0.0238 g, 0.1 mmol), 1,2-bis(4-carboxylatopyridinium)ethane (0.0434 g, 0.1 mmol), NaN_3_ (0.052 g, 0.8 mmol) and distilled water (3 ml) were placed into the bomb and sealed. The bomb was then heated in a 70°C oven for 3 d and allowed to cool to room temperature. The clear colorless solution was decanted to give sheet orange crystals. Yield: 71.7%. Elemental analysis: calculated for C_14_H_24_CoN_8_O_10_: C 32.13, H 4.62, N 21.41%; found: C 32.28, H 4.79, N 21.73%. IR (KBr, ν/cm^-1^): 2086, 1607, 1561, 1457, 1372, 1193, 1138, 1110, 1043, 782, 686.

### Refinement

All hydrogen atoms attached to carbon atoms were placed at calculated positions and refined with the riding model using AFIX 43 and AFIX 23 instructions for aromatic C—H and secondary CH_2_. The water hydrogen atoms were initially located from difference Fourier maps and refined isotropically with restraints on O—H distance (0.85 Å) and H—O—H angle, and *U*_iso_(H) = 1.5*U*_eq_(O). The free water molecule is disordered over two positions with the occupancies being refined to be 0.79 (O5) and 0.21 (O5').

### Crystal data

**Table:**

[Co(N_3_)_2_(H_2_O)_4_]·C_14_H_12_N_2_O_4_·2H_2_O	*Z* = 1
*M**_r_* = 523.34	*F*(000) = 271
Triclinic, *P*1	*D*_x_ = 1.655 Mg m^−^^3^
Hall symbol: -P 1	Mo *K*α radiation, λ = 0.71073 Å
*a* = 7.1951 (5) Å	Cell parameters from 7436 reflections
*b* = 9.0354 (7) Å	θ = 2.5–27.6°
*c* = 9.0915 (5) Å	µ = 0.89 mm^−^^1^
α = 71.402 (3)°	*T* = 296 K
β = 85.568 (2)°	Sheet, orange
γ = 69.752 (2)°	0.08 × 0.08 × 0.02 mm
*V* = 525.20 (6) Å^3^	

### Data collection

**Table:**

Bruker APEXII CCD area-detector diffractometer	2029 independent reflections
Radiation source: fine-focus sealed tube	2016 reflections with *I* > 2σ(*I*)
graphite	*R*_int_ = 0.020
phi and ω scans	θ_max_ = 26.0°, θ_min_ = 2.4°
Absorption correction: multi-scan (*SADABS*; Bruker, 2007)	*h* = −7→8
*T*_min_ = 0.932, *T*_max_ = 0.982	*k* = −11→11
6498 measured reflections	*l* = −11→11

### Refinement

**Table:**

Refinement on *F*^2^	Secondary atom site location: difference Fourier map
Least-squares matrix: full	Hydrogen site location: inferred from neighbouring sites
*R*[*F*^2^ > 2σ(*F*^2^)] = 0.027	H atoms treated by a mixture of independent and constrained refinement
*wR*(*F*^2^) = 0.089	*w* = 1/[σ^2^(*F*_o_^2^) + (0.0517*P*)^2^ + 0.2305*P*] where *P* = (*F*_o_^2^ + 2*F*_c_^2^)/3
*S* = 1.18	(Δ/σ)_max_ < 0.001
2029 reflections	Δρ_max_ = 0.34 e Å^−^^3^
183 parameters	Δρ_min_ = −0.35 e Å^−^^3^
13 restraints	Extinction correction: *SHELXL97* (Sheldrick, 2008), Fc^*^=kFc[1+0.001xFc^2^λ^3^/sin(2θ)]^-1/4^
Primary atom site location: structure-invariant direct methods	Extinction coefficient: 0.072 (7)

### Special details

**Table:**

Geometry. All e.s.d.'s (except the e.s.d. in the dihedral angle between two l.s. planes) are estimated using the full covariance matrix. The cell e.s.d.'s are taken into account individually in the estimation of e.s.d.'s in distances, angles and torsion angles; correlations between e.s.d.'s in cell parameters are only used when they are defined by crystal symmetry. An approximate (isotropic) treatment of cell e.s.d.'s is used for estimating e.s.d.'s involving l.s. planes.
Refinement. Refinement of *F*^2^ against ALL reflections. The weighted *R*-factor *wR* and goodness of fit *S* are based on *F*^2^, conventional *R*-factors *R* are based on *F*, with *F* set to zero for negative *F*^2^. The threshold expression of *F*^2^ > σ(*F*^2^) is used only for calculating *R*-factors(gt) *etc*. and is not relevant to the choice of reflections for refinement. *R*-factors based on *F*^2^ are statistically about twice as large as those based on *F*, and *R*- factors based on ALL data will be even larger.

### Fractional atomic coordinates and isotropic or equivalent isotropic displacement parameters (Å^2^)

**Table:**

	*x*	*y*	*z*	*U*_iso_*/*U*_eq_	Occ. (<1)
Co1	0.5000	0.5000	0.0000	0.02233 (16)	
N1	−0.0182 (2)	0.06625 (19)	0.78289 (17)	0.0240 (3)	
N2	0.5369 (3)	0.4349 (3)	0.2408 (2)	0.0458 (5)	
N3	0.4823 (2)	0.3822 (2)	0.36258 (18)	0.0283 (4)	
O3	0.4820 (2)	0.26543 (17)	0.02090 (16)	0.0300 (3)	
H31	0.425 (4)	0.272 (3)	−0.058 (2)	0.045*	
H32	0.420 (4)	0.229 (3)	0.099 (2)	0.045*	
O4	0.18626 (19)	0.59340 (17)	0.00759 (16)	0.0298 (3)	
H42	0.146 (4)	0.535 (3)	0.087 (2)	0.045*	
H41	0.135 (4)	0.600 (3)	−0.072 (2)	0.045*	
C1	0.1394 (3)	0.2551 (3)	0.3161 (2)	0.0316 (4)	
C2	0.0846 (3)	0.1895 (2)	0.4838 (2)	0.0267 (4)	
C3	0.1985 (3)	0.0345 (2)	0.5774 (2)	0.0284 (4)	
H3A	0.3109	−0.0288	0.5392	0.034*	
C4	0.1448 (3)	−0.0255 (2)	0.7271 (2)	0.0279 (4)	
H4A	0.2211	−0.1298	0.7903	0.033*	
C5	−0.1306 (3)	0.2167 (2)	0.6939 (2)	0.0322 (4)	
H5A	−0.2426	0.2779	0.7342	0.039*	
C6	−0.0818 (3)	0.2812 (3)	0.5435 (2)	0.0335 (4)	
H6A	−0.1602	0.3859	0.4823	0.040*	
C7	−0.0719 (3)	−0.0022 (2)	0.9445 (2)	0.0283 (4)	
H7A	−0.0663	−0.1156	0.9636	0.034*	
H7B	−0.2061	0.0631	0.9607	0.034*	
N4	0.4358 (4)	0.3278 (3)	0.4876 (2)	0.0546 (6)	
O1	0.2747 (3)	0.1567 (2)	0.26605 (18)	0.0470 (4)	
O2	0.0391 (2)	0.4020 (2)	0.24191 (17)	0.0447 (4)	
O5	0.2585 (3)	0.2801 (3)	0.7844 (2)	0.0415 (7)	0.787 (5)
H5	0.344 (3)	0.244 (3)	0.727 (3)	0.062*	
H51	0.193 (5)	0.377 (3)	0.760 (4)	0.062*	0.787 (5)
O5'	0.4261 (18)	0.1544 (12)	0.7953 (9)	0.059 (3)	0.213 (5)
H52	0.506 (13)	0.097 (6)	0.744 (6)	0.089*	0.213 (5)

### Atomic displacement parameters (Å^2^)

**Table:**

	*U*^11^	*U*^22^	*U*^33^	*U*^12^	*U*^13^	*U*^23^
Co1	0.0217 (2)	0.0262 (2)	0.0185 (2)	−0.00944 (14)	0.00190 (12)	−0.00503 (13)
N1	0.0264 (7)	0.0293 (8)	0.0161 (7)	−0.0115 (6)	0.0029 (5)	−0.0050 (6)
N2	0.0542 (12)	0.0691 (13)	0.0221 (9)	−0.0363 (11)	0.0023 (8)	−0.0080 (8)
N3	0.0273 (8)	0.0341 (8)	0.0252 (9)	−0.0110 (7)	−0.0001 (6)	−0.0105 (7)
O3	0.0309 (7)	0.0337 (7)	0.0279 (7)	−0.0147 (6)	0.0038 (5)	−0.0096 (6)
O4	0.0256 (6)	0.0341 (7)	0.0267 (7)	−0.0118 (5)	0.0019 (5)	−0.0041 (6)
C1	0.0371 (10)	0.0469 (11)	0.0172 (8)	−0.0266 (9)	−0.0001 (7)	−0.0048 (8)
C2	0.0309 (9)	0.0378 (10)	0.0169 (8)	−0.0208 (8)	−0.0002 (7)	−0.0058 (7)
C3	0.0308 (9)	0.0330 (9)	0.0230 (9)	−0.0120 (8)	0.0068 (7)	−0.0108 (7)
C4	0.0299 (9)	0.0262 (8)	0.0231 (8)	−0.0073 (7)	0.0028 (7)	−0.0048 (7)
C5	0.0291 (9)	0.0329 (10)	0.0260 (9)	−0.0043 (8)	0.0027 (7)	−0.0052 (8)
C6	0.0327 (10)	0.0333 (10)	0.0248 (9)	−0.0077 (8)	−0.0026 (7)	0.0008 (8)
C7	0.0322 (9)	0.0345 (10)	0.0163 (8)	−0.0142 (8)	0.0062 (7)	−0.0037 (7)
N4	0.0637 (14)	0.0823 (16)	0.0274 (10)	−0.0407 (13)	0.0125 (9)	−0.0150 (10)
O1	0.0690 (11)	0.0510 (9)	0.0277 (7)	−0.0306 (9)	0.0198 (7)	−0.0143 (7)
O2	0.0378 (8)	0.0585 (10)	0.0247 (7)	−0.0191 (7)	0.0001 (6)	0.0074 (7)
O5	0.0513 (14)	0.0524 (13)	0.0298 (10)	−0.0267 (11)	0.0064 (8)	−0.0162 (9)
O5'	0.107 (9)	0.048 (5)	0.029 (4)	−0.031 (5)	−0.014 (4)	−0.011 (3)

### Geometric parameters (Å, °)

**Table:**

Co1—N2	2.0903 (18)	C1—C2	1.524 (2)
Co1—N2^i^	2.0903 (18)	C2—C3	1.382 (3)
Co1—O3^i^	2.1152 (14)	C2—C6	1.384 (3)
Co1—O3	2.1152 (14)	C3—C4	1.375 (3)
Co1—O4^i^	2.1230 (13)	C3—H3A	0.9300
Co1—O4	2.1230 (13)	C4—H4A	0.9300
N1—C5	1.340 (2)	C5—C6	1.376 (3)
N1—C4	1.350 (2)	C5—H5A	0.9300
N1—C7	1.479 (2)	C6—H6A	0.9300
N2—N3	1.154 (2)	C7—C7^ii^	1.519 (4)
N3—N4	1.159 (3)	C7—H7A	0.9700
O3—H31	0.826 (16)	C7—H7B	0.9700
O3—H32	0.847 (16)	O5—H5	0.824 (17)
O4—H42	0.846 (16)	O5—H51	0.802 (17)
O4—H41	0.812 (16)	O5'—H5	0.894 (17)
C1—O1	1.239 (3)	O5'—H52	0.85 (2)
C1—O2	1.256 (3)		
			
N2—Co1—N2^i^	180.0	O1—C1—O2	126.83 (18)
N2—Co1—O3^i^	89.06 (7)	O1—C1—C2	116.63 (18)
N2^i^—Co1—O3^i^	90.94 (7)	O2—C1—C2	116.51 (18)
N2—Co1—O3	90.94 (7)	C3—C2—C6	118.98 (16)
N2^i^—Co1—O3	89.06 (7)	C3—C2—C1	120.08 (18)
O3^i^—Co1—O3	180.00 (8)	C6—C2—C1	120.92 (18)
N2—Co1—O4^i^	87.27 (7)	C4—C3—C2	119.67 (17)
N2^i^—Co1—O4^i^	92.73 (7)	C4—C3—H3A	120.2
O3^i^—Co1—O4^i^	88.65 (5)	C2—C3—H3A	120.2
O3—Co1—O4^i^	91.35 (5)	N1—C4—C3	120.32 (17)
N2—Co1—O4	92.73 (7)	N1—C4—H4A	119.8
N2^i^—Co1—O4	87.27 (7)	C3—C4—H4A	119.8
O3^i^—Co1—O4	91.35 (5)	N1—C5—C6	120.57 (18)
O3—Co1—O4	88.65 (5)	N1—C5—H5A	119.7
O4^i^—Co1—O4	180.00 (3)	C6—C5—H5A	119.7
C5—N1—C4	120.90 (15)	C5—C6—C2	119.56 (18)
C5—N1—C7	120.17 (15)	C5—C6—H6A	120.2
C4—N1—C7	118.94 (15)	C2—C6—H6A	120.2
N3—N2—Co1	148.27 (17)	N1—C7—C7^ii^	109.17 (18)
N2—N3—N4	177.0 (2)	N1—C7—H7A	109.8
Co1—O3—H31	109.2 (19)	C7^ii^—C7—H7A	109.8
Co1—O3—H32	113.4 (18)	N1—C7—H7B	109.8
H31—O3—H32	108 (2)	C7^ii^—C7—H7B	109.8
Co1—O4—H42	111.1 (18)	H7A—C7—H7B	108.3
Co1—O4—H41	110.5 (19)	H5—O5—H51	121 (3)
H42—O4—H41	112 (2)	H5—O5'—H52	107 (3)
			
O3^i^—Co1—N2—N3	129.7 (4)	C5—N1—C4—C3	0.1 (3)
O3—Co1—N2—N3	−50.3 (4)	C7—N1—C4—C3	179.88 (17)
O4^i^—Co1—N2—N3	−141.6 (4)	C2—C3—C4—N1	−0.1 (3)
O4—Co1—N2—N3	38.4 (4)	C4—N1—C5—C6	−0.2 (3)
O1—C1—C2—C3	7.4 (3)	C7—N1—C5—C6	−179.94 (18)
O2—C1—C2—C3	−174.40 (18)	N1—C5—C6—C2	0.2 (3)
O1—C1—C2—C6	−170.78 (19)	C3—C2—C6—C5	−0.1 (3)
O2—C1—C2—C6	7.4 (3)	C1—C2—C6—C5	178.15 (18)
C6—C2—C3—C4	0.0 (3)	C5—N1—C7—C7^ii^	−107.1 (2)
C1—C2—C3—C4	−178.22 (17)	C4—N1—C7—C7^ii^	73.1 (3)

Symmetry codes: (i) −*x*+1, −*y*+1, −*z*; (ii) −*x*, −*y*, −*z*+2.

### Hydrogen-bond geometry (Å, °)

**Table:**

*D*—H···*A*	*D*—H	H···*A*	*D*···*A*	*D*—H···*A*
O3—H31···O5^iii^	0.83 (2)	1.91 (2)	2.727 (2)	170 (2)
O3—H31···O5'^iii^	0.83 (2)	1.96 (2)	2.664 (8)	143 (3)
O4—H41···O2^iv^	0.81 (2)	2.07 (2)	2.870 (2)	167 (3)
O5—H51···O2^v^	0.80 (2)	2.11 (2)	2.877 (3)	160 (4)
O3—H32···O1	0.85 (2)	1.85 (2)	2.694 (2)	178 (3)
O4—H42···O2	0.85 (2)	1.85 (2)	2.687 (2)	172 (2)
O5—H5···N4	0.82 (2)	2.19 (2)	2.864 (3)	139 (2)

Symmetry codes: (iii) *x*, *y*, *z*−1; (iv) −*x*, −*y*+1, −*z*; (v) −*x*, −*y*+1, −*z*+1.
